# High care and low overprotection from both paternal and maternal parents predict a secure attachment style with a partner among perinatal Japanese women

**DOI:** 10.1038/s41598-023-42674-1

**Published:** 2023-09-21

**Authors:** Ekachaeryanti Zain, Naoki Fukui, Yuichiro Watanabe, Koyo Hashijiri, Takaharu Motegi, Maki Ogawa, Jun Egawa, Toshiyuki Someya

**Affiliations:** 1 Department of Psychiatry, Niigata University Graduate School of Medical and Dental Sciences, 757 Asahimachidori-ichibancho, Chuo-ku, Niigata, 951-8510 Japan; 2 Department of Psychiatry, Faculty of Medicine, Mulawarman University, Samarinda, Indonesia

**Keywords:** Epidemiology, Psychiatric disorders

## Abstract

This study aimed to determine how paternal and maternal parenting before adolescence affects adult attachment to a partner during the perinatal period, using three different models of attachment. We used the Parental Bonding Instrument (PBI) and the Relationship Questionnaire (RQ) to examine perceived parenting practices and adult attachment styles, respectively. The participants included 4586 Japanese women who were pregnant or who had given birth, up until one month after childbirth. We performed structural equation modeling analysis between PBI and RQ scores with three different category models, including the four-category model (secure, fearful, preoccupied, and dismissive attachment) as Model 1, the two-category model (model of the self and others) as Model 2, and the single-category model (total attachment style) as Model 3. Models 1 and 2 showed a good fit. Both path models showed a significant association between adult attachment style and perceived paternal and maternal parenting before adolescence, where high care and low overprotection from both paternal and maternal parents predicted adult attachment. Our findings indicate that attachment styles are best described using the four-category and two-category models, and suggest that both paternal and maternal overprotection and care influence adult attachment with a partner during the perinatal period.

## Introduction

The adult attachment style of a pregnant woman, specifically, attachment to a romantic partner, is an essential factor in mentally preparing for the transformation in self-identity from a person who receives care to one who provides care as a parent^1^. In addition, the quality of the maternal–fetal relationship, which relies on the care provider, is associated with a pregnant woman’s attachment style to her partner^2^. Furthermore, attachment can influence how an expectant mother builds an emotional relationship, bonds, and attaches to the fetus^3^. Thus, the adult attachment style affects the mental well-being of mothers and fetuses during the perinatal period, and continues to have an influence during the formation of antenatal attachment to the newborn. For instance, a mother with insecure adult attachment to her partner may perceive her infant’s temperament as more difficult compared with a mother with secure attachment. This could be caused by a less caring and less supportive attitude toward children^4^. An insecure adult attachment style with respect to one’s partner has been related to antenatal and postnatal depression^5,6^, maternal separation anxiety^7^, difficulties regulating emotions^8^, increased vulnerability to stress^9^, greater parenting stress^10,11^, greater levels of suicidal ideation, and more frequent suicide attempts in women during the perinatal period^12^. In contrast, individuals with a secure attachment style appear to have better mental well-being and parenting motivation, and to provide better parental care^13–15^.

According to the former attachment theory developed by Bowlby^16,17^, “the infant and young child should experience a warm, intimate, and continuous relationship with his mother (or permanent mother substitute) in which both find satisfaction and enjoyment” to develop an adult attachment style that is secure. Furthermore, the relationship between a child and parent contributes to the formation of internal representations or working models of the self in relation to others, which determine one’s attachment style. The self-model represents one’s self-worthiness or self-reliance, and the other-model represents the availability or the need for closeness and presence of others in a relationship. This is known as the two-category model of adult attachment style. A secure attachment style is characterized by both a positive self-model and a positive other-model, such that one is self-assured while simultaneously valuing relationships with others^18^.

Bartholomew and Horowitz expanded the two-category attachment model proposed by Bowlby into a four-category model, which includes secure, fearful, preoccupied, and dismissive attachment styles^19^. They also developed the Relationship Questionnaire (RQ) to measure adult attachment styles to a partner^19^. Individuals with secure attachment styles are comfortable with autonomy and intimacy, while individuals with fearful attachment styles are dependent and socially avoidant. Individuals with preoccupied attachment styles show strong dependency on others and are preoccupied with intimacy. Meanwhile, individuals with dismissive attachment styles are counter-dependent and avoidant of intimacy. The last three styles are considered to be forms of insecure attachment.

Adult attachment has been associated with various factors, such as the quality of an individual’s childhood caregiving environment (e.g., maternal depression, father absence), the perceived quality of the parenting they received during childhood, their social competence, and the quality of their best friendships throughout their lifetime^20^. In this study, we focused on the perceived quality of parenting before adolescence, as evaluated retrospectively by expectant mothers. According to the Parental Bonding Instrument (PBI)^21^, perceived parenting practices can be categorized into two factors: care and overprotection. Parental care is defined as the provision of affection, emotional warmth, and autonomy, whereas parental overprotection is characterized by emotional coldness, control, and interference. A previous study suggested that a secure attachment style in women can be predicted by low levels of maternal overprotection and high levels of maternal and paternal care^22^. Another prospective cohort study similarly reported that perceptions of optimal maternal parenting, i.e., high care and low control from one’s mother, contributed to secure adult attachment in women 30 years after an initial assessment of perceived parenting^23^. In addition, higher perceived levels of paternal care in women resulted in a more positive self-image^23^. These findings highlight the importance of both paternal and maternal parenting style, and suggest that parenting style is strongly associated with attachment formation throughout the lifespan. However, no studies have examined whether paternal overprotection significantly predicts secure attachment in female offspring. Furthermore, the way in which attachment style influences women in the perinatal period, which includes the beginning of pregnancy and extends to the postpartum period, is unknown. Perinatal women are considered to be a vulnerable population requiring specialized mental health care because of the significant potential consequences of perinatal mental illness^24^. Therefore, understanding the nature of adult attachment and the contribution of perceived parenting practices according to parental gender could advance perinatal mental healthcare.

Previous studies have reported causal models with a good fit to the two-category^14^ and four-category^22^ models of adult attachment style in relation to perceived parenting style. However, no studies have examined this topic using a single-category model of total attachment style (TAS)^25^ with a path model analysis. In addition, all previous studies used a model that combined the paternal and maternal PBI data in the latent variable of the model. Our previous study indicated that parenting by one’s mother, as opposed to their father, might be more important for maintaining satisfactory maternal-fetus bonding in women^26^. Among new mothers, the effects of parenting by their mothers might differ from that of their fathers according to specific life events and mental health status. To address this in the present study, we analyzed PBI data regarding the fathers and mothers of new mothers using a path model in which the data from the two parents were not combined as the latent variable.

The goal of this study was to determine how perceived pre-adolescence paternal and maternal parenting affected adult attachment style using three attachment models. Prior to performing path analyses, we formulated the following hypotheses regarding the expected relationships among the variables. First, we hypothesized that the best-fitting path model would result in a reliable correlation between adult attachment style and perceived paternal and maternal parenting. Second, we hypothesized that high levels of paternal care and low levels of paternal overprotection would predict a secure attachment style, which is characterized by high secure and low fearful, preoccupied, and dismissive attachment styles in Model 1, by a high positive self-model and high positive other-model in Model 2, and by a high TAS in Model 3. Third, we hypothesized that high levels of maternal care and low levels of maternal overprotection would similarly predict the abovementioned relationships.

## Methods

### Ethics statement

This study involving human subjects followed the principles of the Declaration of Helsinki. We obtained approval from the ethics committee of Niigata University (Approval Number: 2016–0019) and the ethics committees of the participating obstetric institutions. We obtained written informed consent from all participants.

### Participants

This study was part of the Perinatal Mental Health Research Project^26–31^, which included the Department of Obstetrics and Gynecology at Niigata University Medical and Dental Hospital as well as 33 associated obstetric institutions in Niigata prefecture, Japan. Between March 2017 and March 2021, we distributed a large-scale self-report questionnaire survey to pregnant Japanese women aged 18 years and older. The exclusion criteria were serious physical complications, serious pregnancy complications, or severe psychiatric disorders (e.g., severe schizophrenia or severe depression). Participants completed the RQ and PBI questionnaires. Some participants were from the same dataset as our previous study, which found that perceived negative parenting before adolescence had direct and indirect effects (via anxiety and depression) on maternal-infant bonding in the perinatal period in 1301 pregnant Japanese women^26^.

### Measures

The RQ measures adult attachment style in a relationship with a partner^19^. It measures four adult attachment styles: secure, fearful, preoccupied, and dismissive. Each category is described using a paragraph of statements and has a seven-point Likert-type scale ranging from 1 = “Does not apply to me at all” to 7 = “Applies to me very much”. We instructed the participants to choose the number that best described their relationship with their partner. Previous studies have reported the reliability^18^ and validity^19^ of the RQ. We used a Japanese version that was validated previously in Japanese adolescents^25^. There are three different model structures for categorizing and scoring adult attachment styles in the currently available literature. The first is the four-category model (Model 1), which includes the original dimensions of the RQ: secure, fearful, preoccupied, and dismissive attachment^19^. Each category in Model 1 is given a score according to the RQ’s scoring method. The second is the two-category model (Model 2): self-model and other-model. Since we used the RQ, we used the following formula to obtain a score for each category in Model 2: self-model = secure − fearful − preoccupied + dismissive; other-model = secure − fearful + preoccupied − dismissive^14,18^. The formula for scoring was in accordance with the category quadrants in Model 1, where both secure and dismissive attachment styles had a positive quadrant in terms of self-reliance or autonomy, and both secure and preoccupied attachment styles had a positive quadrant in terms of the presence of others in a relationship^19^. Thus, scores for RQ categories that were in a negative quadrant in Model 2 were included as a deduction in the formula. The last is a single-category model (Model 3): total attachment style (TAS) score = secure − fearful − preoccupied − dismissive^25^. This model is formulated from a single bipolar factor representing the secure and insecure attachment styles, where all three insecure attachments are deduction factors.

The PBI measures participants’ subjective experience of the parenting attitude they perceived their parents as having, according to their memory, during their first 16 years^21^. We used two identical instruments, administered independently, to assess perceived paternal and maternal parenting practices. The self-report instrument consisted of 25 items, including 12 items representing parental care and 13 items representing parental overprotection. We instructed the participants to rate each item, which was a statement about a specific parenting behavior, according to how probable that had perceived that behavior to be. They used a four-point Likert-type scale ranging from 0 = “Very unlikely” to 3 = “Very likely”. The PBI categorizes perceived parenting practices into two subscales that we used in this study: care and overprotection. The care subscale measures positive parenting, including items suggesting parental attitudes of care, affection, sensitivity, cooperation, accessibility, and encouragement of autonomy and independence. The overprotection subscale measures negative parenting, including items suggesting parental attitudes of indifference, strictness, punitiveness, rejection, interference, control, and constraint. We used the validated Japanese version of the PBI^32^.

We administered the questionnaires to each participant on separate printed paper forms. We did not change the order of the items, but presented them according to the previously validated version of each original questionnaire. We sequentially grouped the forms in the following order: paternal PBI, maternal PBI, and RQ, in one set. Therefore, we expected that most participants responded to the questionnaires in the order in which they were presented.

### Statistical analyses

We performed structural equation modeling analysis to find correlations between the RQ subscales and PBI subscales. We analyzed the paternal and maternal PBI forms interdependently. The PBI subscales included care and overprotection. The RQ subscales were differentiated into three different models of adult attachment styles, which varied according to the number of categories in the model. Model 1 used a four-category model, which included secure, fearful, preoccupied, and dismissive attachment as the RQ subscales. Model 2 used a two-category model, which included the self-model and other-model. Model 3 used a single-category model, which included the TAS. In Models 1 and 2, we combined the RQ subscales in a latent variable corresponding to attachment style. For each model, we drew paths from each of the PBI subscales of care and overprotection to the latent variable of attachment style. Then, we performed covariance structure analysis on each path model. After these analyses, we retained statistically significant paths (P < 0.05). We adopted the comparative fit index (CFI) and the root mean square error of approximation (RMSEA) as indices of the goodness of fit (CFI ≥ 0.95 and RMSEA ≤ 0.08)^33^ between the models and the data. All statistical analyses were performed using SPSS version 25 (IBM Corp., Armonk, NY, USA) and Amos 25.0.0 (IBM Japan, Tokyo, Japan).

## Results

We included all data from 4586 pregnant women who completed both the RQ and PBI questionnaires, with no missing values (Table 1). We also included questionnaires from pregnant women who had given birth during the course of questionnaire collection, such that they completed the questionnaire up to 1 month after childbirth.

**Table 1:** Characteristics of participants (n = 4586).

Variable	Value
Age (years)	31.94 ± 4.82
Parity (primipara/multipara)	2208/2378
Gestational age (T1/T2/T3)	2995/1238/353
PBI scores	
Paternal care	26.32 ± 7.23
Paternal overprotection	7.37 ± 5.43
Maternal care	30.48 ± 6.02
Maternal overprotection	7.92 ± 6.39
RQ scores	
Model 1	
Secure	4.13 ± 1.62
Fearful	2.82 ± 1.71
Preoccupied	2.65 ± 1.72
Dismissive	2.01 ± 1.34
Model 2	
Self-model	0.67 ± 3.42
Other-model	1.94 ± 3.06
Model 3	
Total attachment style	–3.36 ± 4.33

Data are expressed as mean ± standard deviation.

PBI, Parental Bonding Instrument; RQ, Relationship Questionnaire; T1, 12–15 weeks of pregnancy; T2, 30–34 weeks of pregnancy; T3, 4 weeks after childbirth.

Model 1 and Model 2 met the criteria for a good fit to the data (CFI = 0.990 and 0.995, respectively; RMSEA = 0.036 and 0.046, respectively), whereas Model 3 was not a good fit to the data (CFI = 1.000 and RMSEA = 0.356; Table 2).

**Table 2:** Indicators for the three attachment models.

Indicator	Model 1	Model 2	Model 3
Factor	Secure, fearful, preoccupied, and dismissive	Self- and other-models	Total attachment style
CFI	0.990	0.995	1.000
RMSEA	0.036	0.046	0.356

CFI, comparative fit index; RMSEA, root mean square error of approximation.

**Figure 1:**
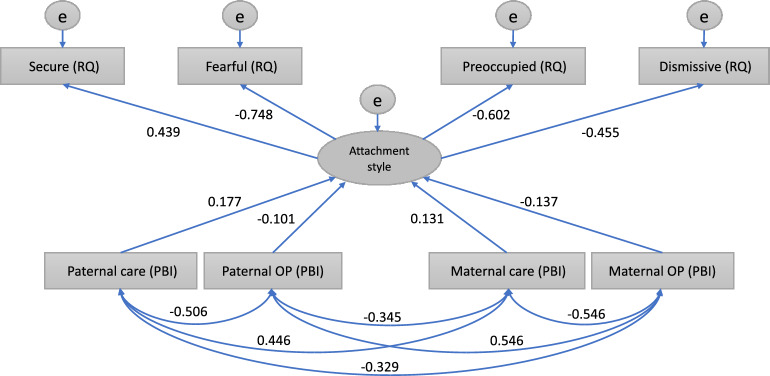
Structural equation modeling for Model 1. Abbreviations: OP, overprotection; PBI, parental bonding instrument; RQ, relationship questionnaire. Secure, fearful, preoccupied, and dismissive attachment styles were the observable variables. Attachment style was a latent variable composed of secure, fearful, preoccupied, and dismissive attachment. Care and OP were observable variables.

In Model 1, paternal care, paternal overprotection, maternal care, and maternal overprotection predicted attachment style (*r* = 0.177, − 0.101, 0.131, and − 0.137, respectively; all *P* < 0.001; Fig. 1). Attachment style included secure, fearful, preoccupied, and dismissive attachment styles (*r* = 0.439, − 0.748, − 0.602, and − 0.455, respectively; all *P* < 0.001).

**Figure 2:**
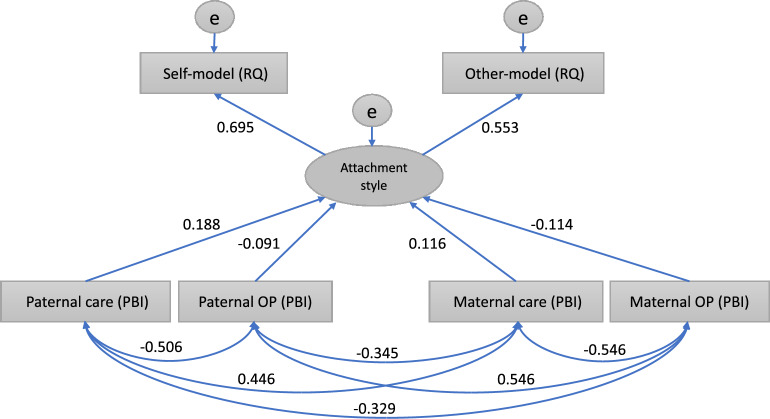
Structural equation modeling for Model 2. Abbreviations: OP, overprotection; PBI, parental bonding instrument; RQ, relationship questionnaire. Self-model = secure − fearful − preoccupied + dismissive , and other-model = secure – fearful + preoccupied − dismissive . Attachment style was a latent variable composed of a self-model and an other-model. Care and OP were observable variables.

In Model 2, paternal care, paternal overprotection, maternal care, and maternal overprotection predicted attachment style (*r* = 0.188, − 0.091, 0.116, and − 0.114, respectively; all *P* < 0.001; Fig. 2). Attachment style included the self-model and the other-model (*r* = 0.695 and 0.553, respectively; all *P* < 0.001).

The correlation coefficients between the RQ subscales of Model 1 and Model 2 are given in Supplementary Tables 1a and 1b.

## Discussion

The present study revealed that both Model 1, which was a four-category model, and Model 2, which was a two-category model, had a path model with good fit. However, Model 3, which was a single-category model, had a bad fit with the dataset. Since SEM is a powerful tool for examining the goodness of fit of a causal model according to both scientific and statistical perspectives^34^, the results of our analysis with Model 1 and Model 2 strongly suggest that adult attachment style is related to perceived paternal and maternal parenting before adolescence. It is essential to apply the correct model for assessing adult attachment. Model 3, previously proposed by Matsuoka et al.^25^, has never been tested via SEM analysis. The present study revealed that a single-category TAS model might not be appropriate for assessing adult attachment style. Furthermore, it may not be possible to accurately assess attachment style using a model with a single category, as it may not be possible to simplify the basic categorization enabled by a two-category or four-category model. Instead, each category may describe a relationship, depending on specific life events and variations across relationships. According to Bowlby^17^, attachment styles are formed by internal working models of interpersonal interactions throughout life, as an individual forms self- and other-models that lead them to seek or avoid various interactions in close relationships. The contributions of both fundamental dimensions define the underlying measures of attachment style in social relationships^18^. We used a SEM analysis to test the compatibility of Model 3 with the four-category model proposed by Bartholomew and Horowitz^19^. Our data show that Model 1 and Model 2 can represent the constructs of the underlying theoretical framework of adult attachment with good reliability and validity, while this was not the case for Model 3.

In this study, the good fit of the two models suggested that there exists a significant association between adult attachment style and perceived paternal and maternal parenting before adolescence. We analyzed parenting by combining the paternal and maternal PBI forms but did not use latent variables of care and overprotection in the models. We included all observable variables of care and overprotection from fathers and mothers, respectively. Previous studies in the Japanese population that involved PBI variables and SEM analysis also combined paternal and maternal parenting in their analyses and models^14,22,26^. Among these, one study combined paternal and maternal parenting in latent variables^14^. However, analyzing the paternal and maternal PBI without combining them in latent variables clarified that perceived parenting from a mother differs from that of a father in terms of the influence on adult attachment style. The present study suggested that both perceived paternal and maternal care are important during the perinatal period in terms of secure attachment to a partner. In that regard, paternal parenting with high levels of care and low levels of overprotection predicted a secure adult attachment style. This was the same for maternal parenting. This finding represents new evidence for how perceived paternal care and overprotection might contribute equally to maternal parenting in terms of forming adult attachment.

Previous studies using SEM analysis found that the latent variable of PBI overprotection was not associated with adult attachment style in 363 Japanese mothers^14^, and that paternal overprotection without a latent variable was not associated with adult attachment style in 2,709 Japanese female university students^22^. In the current study, which had a large sample size, we conducted SEM analysis without latent variables from the PBI subscales of care and overprotection. Our data indicated that both paternal and maternal overprotection and care influenced attachment with a partner in adulthood. However, Shiraishi et al. found that the latent variable of PBI overprotection was not associated with adult attachment style in 363 Japanese mothers^14^. We conducted direct measurements with observable variables to represent the collected scores of our PBI data. This enabled us to test our hypotheses regarding the way in which adult attachment styles were independently associated with paternal care, paternal overprotection, maternal care, and maternal overprotection. The independent associations among these observable variables cannot be directly measured when latent variables are used^35^. The use of a latent variable generally corresponds with hypothetical constructs that have unobservable referents, but their existence can be posited to explain the association among a specified class of observable variables^36^. This may have led to discrepancies between the results of our study and those by Shiraishi et al. Another study that, like our study, used observable variables, reported that maternal but not paternal overprotection was associated with adult attachment style in 2709 Japanese female university students^22^. However, considering that the study populations varied across previous studies, different life phases or events might have influenced perceived parenting throughout the lifespan.

The present study also suggested that during pregnancy, the contribution of perceived paternal care was slightly greater than that for perceived maternal care in forming an adult attachment to a partner. This finding was consistent with a previous study that reported that individuals who were able to form a secure romantic relationship had a father who was caring, loving, humorous, and affectionate^37^. Notably, those study participants tended to describe their opposite-sex parent more favorably than the same-sex parent in terms of caregiver qualities. Another supporting study suggested that perceived paternal care contributed to a positive self-model of adult attachment in women^23^. Our data indicate that in contrast to paternal parenting practices, perceived low maternal overprotection may contribute slightly more than paternal overprotection to the formation of adulthood attachment to one’s partner. As mentioned, a previous study also reported that individuals who could form secure romantic relationships had a mother who was respectful, accepting, not intrusive, and not demanding^37^, which represented the quality of low overprotection. Moreover, participants in the previous study tended to overjudge the negative traits of their same-sex parents^37^. Thus, the relationships with each parent since childhood may separately contribute to the formation of attachment style to a partner in adulthood under specific circumstances. Further studies are needed to characterize the individual effects of paternal and maternal parenting.

In Model 1, the fearful attachment style had a higher load value than the other attachment styles (Fig. 1). This result was in line with previous studies in Japanese populations^22,25^. However, this finding may be a distinctive feature of the Japanese population. A prevalent preoccupied attachment style was found mainly in Asian regions^38^, and this attachment style was also prevalent in the current study, although less prevalent than the fearful style. In contrast, a previous study of 5692 men and women of varying ethnicities, including 72% non-Latino white individuals who provided attachment information using a self-reported questionnaire, found that the attachment styles could be ordered from highest to lowest score as follows: secure, anxious, and avoidant attachment^12^. The anxious dimension corresponds to low self-esteem, emotional closeness, and dependency seeking, whereas the avoidant dimension corresponds to a degree of discomfort with intimacy. According to the four-category model of adult attachment, the fearful style has dimensions of both anxiety and avoidance^19^.

The cross-cultural aspects of attachment styles have been discussed in previous studies^39^. Indeed, attachment styles might be distributed differently across countries because of varying cultural and social systems. However, few studies have explored this^38,39^. According to the attachment theory developed by Ainsworth^40^, individuals develop an attachment type according to their relationships with their mothers as infants. In the ‘strange situation’ test of attachment style, most American infants (70% with standard distribution) were found to have a secure attachment style. In contrast, studies using a similar method found that German infants had a relatively high percentage of avoidant-type attachment^41^, while Japanese infants had a relatively high percentage of resistant-type attachment^42^. The resistant type is defined as dependent behavior with rejection of the attachment figure when they engage in interaction^40^. The avoidant-type of attachment has been examined extensively over the last few years, and is defined as the fearful style in the four-category model^19^. According to the two-category model, the fearful style arises from a combination of a negative view of the self and a negative view of others^19^. However, the present study found high positive loading values for the self-model and other-model, representing the secure attachment style based on Model 2. The emphasis in Asian culture on maintaining a relatively selfless state might explain this, where developing a sense of connectedness to the family and others is encouraged from childhood^43^. In contrast, developing a sense of self might be more emphasized in European culture^43^. In addition, demands with a high potential for emotional conflict in romantic relationships may differ across cultures^39^. Japanese individuals might expect fewer emotional conflicts, such that conflict defines emotional closeness in their relationships with others. Further, the expectation of rejection from others may lead Japanese individuals to avoid intimacy^23^. In this regard, although the fearful attachment style in the present study had a higher loading value in Model 1, there was some dissonance associated with this attachment style in Model 2. This discrepancy may be explained by the results of a study on romantic attachment, conducted in 62 cultural regions^38^. The study reported a strongly significant negative correlation between the secure and fearful subscales of the RQ in Western regions, while this correlation was non-significant in some African and most Asian regions, including Japan^38^. Therefore, the combination of a negative self-model and a negative other-model might not underlie a fearful attachment style in Asian or specifically Japanese populations. Further studies are needed to construct a normative form of the secure attachment style in an Asian population.

Our study has several limitations that merit discussion. First, we did not collect data on the participants’ family-structure history or related adverse life events such as a history of abuse or attachment figure separations from childhood to adulthood. Moreover, we did not consider data about the parental figures involved in parenting, such as mother-father pairs, single mothers, single fathers, and substitute parental figures. Therefore, we could not evaluate the differences in perceived parenting among groups that were stratified using these data. Although we performed our data analysis using data for which both the paternal and maternal PBI were available, the generalizability of various family-structure histories may influence our findings. Second, we did not examine data on the current marital status of the participants. In addition, we did not collect data on the partner’s attachment style in the current romantic relationship. Since we focused on finding the best model for use in future perinatal psychiatry studies, we only targeted women. Accordingly, we evaluated both perceived maternal and paternal parenting styles using PBI only in women participants. We did not evaluate the parenting style of their male partners, as this was beyond the scope of our study. Furthermore, we did not examine data regarding the relationship circumstances in the current family, nor that associated with current stressful life events. Therefore, we could not evaluate the effects of these potential contributing or confounding variables on adult attachment styles. Finally, we did not balance the order of the paternal and maternal PBI. Therefore, we cannot speculate regarding the influence of the order of presentation of the paternal and maternal PBI questionnaires.

In conclusion, the present study showed that attachment styles are best described using four-category and two-category models. These models suggested a significant association between adult attachment style and perceived paternal and maternal parenting before adolescence, where high care and low overprotection from both paternal and maternal parents predicted a secure attachment style. Our findings support the use of attachment models when providing perinatal care, particularly in preparing parents for caregiver roles starting from early pregnancy. However, further studies on the longitudinal implications of parenting practices in terms of the development of attachment styles are needed to clarify the consistency of these effects throughout the lifespan. It is also essential to determine secure attachment styles and positive parenting practices that are normative in Asian populations.

### Supplementary Information

Supplementary Information.

## Data Availability

All relevant data are provided in the paper. We are not able to make the underlying data available to readers, because we do not have permission from the participating institutions to do so.
