# Supplementary material for: High care and low overprotection from both paternal and maternal parents predict a secure attachment style with a partner among perinatal Japanese women
Source: Sci Rep. 2023 Sep 21;13:15684. doi: 10.1038/s41598-023-42674-1 (PMC10514324; doi:10.1038/s41598-023-42674-1)
Supplement: Supplementary file 1 — Supplementary Information. [file 41598_2023_42674_MOESM1_ESM.docx]

**Supplementary Tables**

**Supplementary table 1a**. Correlation coefficients for Relationship Questionnaire (RQ) subscales of four-category model (Model 1)

|  | **1** | **2** | **3** | **4** |
| --- | --- | --- | --- | --- |
| 1. Secure |  |  |  |  |
| 2. Fearful | -.319* |  |  |  |
| 3. Preoccupied | -.265* | .461* |  |  |
| 4. Dismissing | -.227* | .351* | .223* |  |
| **Mean** | 4.13 | 2.82 | 2.65 | 2.01 |
| **SD** | 1.62 | 1.71 | 1.55 | 1.34 |

Abbreviations: RQ, Relationship Questionnaire; PBI, Parental Bonding Instrument; SD, standard deviation.

*Level of significance set at P < 0.0083 according to the Bonferroni correction of 6 statistical tests

**Supplementary table 1b**. Correlation coefficients for Relationship Questionnaire (RQ) subscales of two-category model (Model 2)

|  | **1** | **2** |
| --- | --- | --- |
| 1. Self-model |  |  |
| 2. Other-model | .348* |  |
| **Mean** | 0.67 | 1.94 |
| **SD** | 3.42 | 3.06 |

Abbreviations: RQ, Relationship Questionnaire; PBI, Parental Bonding Instrument; SD, standard deviation.

*Level of significance set at P < 0.05
